# Dose–response relationship between physical activity and cardiometabolic risk in obese children and adolescents: A pre-post quasi-experimental study

**DOI:** 10.3389/fphys.2023.1070653

**Published:** 2023-01-19

**Authors:** Zekai Chen, Lin Zhu

**Affiliations:** ^1^ Graduate School, Guangzhou Sport University, Guangzhou, China; ^2^ School of Sport and Health, Guangzhou Sport University, Guangzhou, China

**Keywords:** physical activity, cardiometabolic risk, obesity, dose-response relationship, children and adolescents

## Abstract

**Objective:** This study aims to explore the dose-response relationship between the daily duration of moderate to vigorous physical activity and the improvement of cardiometabolic risk indicators in obese children and adolescents.

**Methods:** Seventy-seven obese children and adolescents aged 10–17 years were randomly recruited for a 4-week exercise intervention in a closed camp during 2019–2021, physical activity was monitored by ActiGraph GT3X + to obtain daily MVPA duration, and the improvement of CMR indicators were reflected by the changes (Δ) of waist circumference, systolic blood pressure, diastolic blood pressure, total cholesterol, triglyceride , high-density lipoprotein cholesterol, low-density lipoprotein cholesterol, fasting insulin, fasting plasma glucose, and homeostasis model assessment of insulin resistance before and after the intervention, calculated as ‘‘Δ+indicator” = values after intervention–values before intervention. The groups were divided into different doses of Q1∼Q3 according to the daily MVPA duration from low to high. The differences in the improvement of different dose groups were compared by one-way analysis of covariance, and the dose-response relationship between MVPA duration and CMR indicators improvement was analyzed by linear regression and piecewise regression. The nonlinear relationship was analyzed by restricted cubic spline.

**Results:** 1) Compared with indicators before the intervention, WC, SBP, DBP, TC, TG, HDL-C, LDL-C, FINS, and HOMA-IR were significantly lower after the intervention (*p*-value < 0.05). 2) The dose-response relationship between MVPA and LDL-C improvement was non-linear (*P*-Nonlinear < 0.05). When MVPA >77.1min/day, ΔLDL-C further decreased with the increase of MVPA duration [*β* = −0.009, 95% confidence interval (CI): −0.013, −0.005], and when MVPA ≤77.1min/day, increasing the MVPA duration did not increase the improvement of ΔLDL-C.

**Conclusion:** There was a nonlinear dose-response relationship between the daily MVPA duration and LDL-C improvement in obese children and adolescents. In order to obtain more significant improvement in LDL-C through increased MVPA duration, MVPA duration should be higher than 77.1 min/day.

## 1 Introduction

The incidence of childhood and adolescent obesity has been on the rise globally over the last half-century, and this has placed a huge burden on the current global public health system (Afshin et al., 2017). As of 2017, the detection rate of obesity among children and adolescents aged 9–17 years in China has exceeded 10% (Zhu et al., 2019), while globally, more than 100 million children and adolescents have obesity problems, and if no effective measures are taken, the total number of overweight and obese children worldwide is expected to exceed 250 million (Shah et al., 2020). Obesity can lead to an increased risk of heart disease by aggregating cardiometabolic risk (CMR) factors such as hypertension, insulin resistance, dyslipidemia, and type 2 diabetes (Chung et al., 2018). In addition, the development of obesity in childhood and adolescence can have a profound negative impact on physical health status in adulthood (Suglia et al., 2018), and evidence from cross-sectional and prospective studies suggests that obesity in childhood and adolescence is associated with the development of atherosclerosis in adulthood (Berenson et al., 1998; Cote et al., 2015). The significance of early intervention in obese children and adolescents to improve cardiometabolic health cannot be overstated.

Physical activity (PA) not only improves the physical shape of obese children and adolescents but also its effect on cardiometabolic health has been confirmed by previous studies (Väistö et al., 2014; Júdice et al., 2020). The correlation between moderate to vigorous physical activity (MVPA) and improved cardiometabolic health is considered to be high, while the correlation between light physical activity (LPA) and improved cardiometabolic health is negligible (Hansen et al., 2018; Väistö et al., 2019). Current physical activity guidelines issued by national and international authorities recommend that children and adolescents should engage in MVPA for at least 60 min per day to achieve significant health benefits (Misra et al., 2012; Kahlmeier et al., 2015; Zhan et al., 2017; Piercy et al., 2018; Bull et al., 2020; Clutterbuck and Johnston, 2021), which gives children and adolescents the basis for exercising in their daily lives. Obese children and adolescents are at higher risk of injury when exercising than normal-weight ones, and to balance safety and efficiency, exercise needs to be planned precisely according to the dose-response relationship between physical activity and health effects, thus ensuring higher health improvement by increasing physical activity within a reasonable range. However, existing physical activity guidelines and related studies have not clarified the quantitative dose-response relationship between MVPA duration and cardiometabolic health improvement in obese children and adolescents. Therefore, this study tried to investigate the dose-response relationship between physical activity and CMR indicators in obese children and adolescents by monitoring and evaluating the effect of physical activity and CMR indicators and analyze whether there was a non-linear relationship and threshold effect, aiming to provide a scientific and accurate theoretical basis for improving cardiometabolic health through exercise in obese children and adolescents.

## 2 Materials and methods

### 2.1 Study participants

Participants were randomly recruited between 2019 and 2021 through the Shenzhen Weight Watchers Boot Camp. Inclusion criteria: 1) complying with the obesity criteria in the National Health and Family Planning Commission (NHFPC) WS/T586-2018 (for both boys and girls aged 6–17 years, overweight was defined as BMI ≥85th percentile whereas obesity was defined as BMI ≥95th percentile, stratified by gender and age. For example, for 10-year-old boys, BMI ≥19.2 or higher was considered overweight and BMI ≥21.9 was considered obese, whereas for 11-year-old boys, the BMI cut-off points for overweight and obesity were 19.9 and 23.0 respectively, the specific BMI cut-off points depend on age and gender, further details are provided in the supplementary material.); 2) aged 10–17 years; 3) participant’s parents or guardians were informed of the study and signed an informed consent form. Exclusion criteria: 1) those who were taking medication for obesity; 2) those with cardiopulmonary insufficiency and exercise limitation; 3) those who were overweight or normal weight status. Finally, a total of 77 participants were included.

### 2.2 Data collection and procedure

#### 2.2.1 Anthropometric measurement

The height and weight of the participants were measured in centimeters (cm) and kilograms (kg), respectively, using the equipment specified for national physical fitness monitoring, and the results were accurate to one decimal point. Body mass index (BMI) was calculated from the measured height and weight, with BMI = weight (kg)/height (m^2^). Waist circumference (WC) was measured using a leather tape measure, and the test method was performed according to the requirements of National Health and Family Planning Commission (NHFPC) WS/T611-2018.

#### 2.2.2 Blood pressure measurement

The systolic blood pressure (SBP) and diastolic blood pressure (DBP) of the participants were measured using a Omron electronic blood pressure monitor (OMRON HEM-1020, China), and the test was performed according to the requirements of National Health and Family Planning Commission (NHFPC) WS/T610-2018.

#### 2.2.3 Physical activity measurement

Participants’ physical activity during camp was recorded by wearing an ActiGraph GT3X + triaxial motion accelerometer (ActiGraph, Pensacola, FL, United States), which has been shown to be applicable to physical activity measurements in children and adolescents (Mora-Gonzalez et al., 2019; Mayorga-Vega et al., 2021). All participants were asked to wear the accelerometer at the intersection of the midline of the right axilla and the horizontal line of the iliac spine (Xing et al., 2021). Testers set the sampling frequency of 30Hz and the sampling interval of the 60s in advance, and the participants wore the accelerometer 6 days a week for 4 weeks and were not allowed to remove it except for activities such as sleeping and bathing and swimming, and it was effectively worn for at least 8 h a day (Cain et al., 2013; Rich et al., 2013; Migueles et al., 2017). The accelerometers were distributed by the testers at 09:00 each morning and collected at 21:00 that evening, and the instrument was set to record data during that period for 12 h. Data were recorded, exported, and analyzed by using ActiLife Version 6.13.3 software, and the period in which no physical activity data were monitored for 60 min in a row was set as not effectively wearing the accelerometer before exporting the analysis (Skrede et al., 2017), and participants had at least 2 weeks and ≥3 days per week of effective wearing days before being included in the analysis. The cut point for MVPA selected for this study was the cut point criterion previously established by our team for obese children and adolescents, and VM_3_ ≥ 3,687 counts/min was determined to be MVPA (Liu et al., 2021). Participants performed 4 h of aerobic exercise based training sessions with a coach each day, 2 h in the morning and 2 h in the afternoon, participants were free to engage in physical activity outside of training sessions, 6 days per week, for a total of 4 weeks.

#### 2.2.4 Cardiometabolic risk indicator measurement

The participants blood samples were collected at the designated hospital at 8:00 a.m. on the second day of the camp, and all participants fasted for at least 12 h before the blood collection. Total cholesterol (TC), triglyceride (TG), high density lipoprotein cholesterol (HDL-C), low density lipoprotein (LDL-C), were measured using a fully automated biochemical analyzer (AU5800, Beckman, Japan). Fasting plasma glucose (FPG) was measured by the glucose oxidase method, and fasting insulin (FINS) was measured by electrochemiluminescence immunoassay. The homeostasis model assessment of insulin resistance (HOMA-IR) = FPG (mmol/L) × FINS (mIU/L)/22.5 (Matthews et al., 1985).

#### 2.2.5 Resting energy expenditure test

The resting energy consumption of the participants was obtained by indirect calorimetry, which is considered the gold standard for energy consumption testing (Boullata et al., 2007). The Cortex Meta Max 3B portable gas metabolism analyzer was used to measure the resting energy consumption of the participants. All participants completed the resting energy test within 1 week before the official start of exercise training and were asked to refrain from drinking coffee and alcoholic beverages for 24 h before the test and to avoid the influence of food-specific kinetic effects on the test results by having an interval of more than 2 h between the official test and the meal. During the test, the participant was sedentary for 10–15 min in a stable room temperature test site, and then the tester put on the apparatus and performed the resting energy test for 15 min in a quiet and lying position, during which the participant was awake. The amount of O_2_ inhaled (mL/min) and CO_2_ exhaled (mL/min) in each breath were recorded, and then the resting energy consumption was calculated according to the formula of weir (Weir, 1949).

#### 2.2.6 Diet plan

All participants were put on a diet by a professional dietitian hired by the camp, and the daily caloric intake through diet was calculated based on the resting energy expenditure (REE) of the participants.REE (kcal/min) = 3.9*VO_2_(L/min)+1.1*VCO_2_(L/min); REE (kcal/day) = REE (kcal/min)*1,440. In order to match energy expenditure with REE, nutrition experts designed the diet plan in accordance with the Chinese Food Composition Table compiled by the Chinese Center for Disease and Prevention. The ratio of calorie distribution among the three meals a day was approximately 3:4:3, with the proportion of carbohydrate, protein, and fat supplying 55%–65%, 10%–15%, and 20%–35% of the total energy of each meal, respectively. The main types of food included cereals, meat, eggs, vegetables, and fruits.

#### 2.2.7 Statistical analysis

Continuous variables were expressed as mean ± standard deviation ( $\bar{x}$ ±s) or median and interquartile range (IQR), and the normality of the data distribution was tested before formal analysis, and then parametric or nonparametric statistical methods were selected for analysis based on the results. A paired-sample *t*-test or rank-sum test was used to compare the changes of each indicator before and after the intervention. Partial *η* ^2^ were used to reflect the effect size, 0.01 ≤ Partial *η* ^2^ < 0.06 is for small effects, 0.06 ≤ Partial *η* ^2^ < 0.14 is for medium effects and Partial *η* ^2^ > 0.14 is for large effects. The improvement of each cardiometabolic indicator was expressed by the change quantity “Δ+indicator”, as calculated by value after intervention minus value before the intervention, where a positive value of Δ for HDL-C indicates improvement, and a negative value of Δ for all indicators except HDL-C indicates improvement. The dose groups (Q1∼Q3) were divided into low to high dose groups according to the daily MVPA duration using the trichotomous method, and the differences in improvement between the Q1∼Q3 groups were compared using a one-way analysis of covariance (ANCOVA), with *post hoc* analysis using the Bonferroni method, baseline indicator values as covariates. Linear regression was used to explore the dose-response relationship between each 1 min/day increase in MVPA and the improvement in each indicator. Restricted cubic spline (RCS) (Desquilbet and Mariotti, 2010) was used to explore whether there was a nonlinear relationship between the daily MVPA duration and the improvement in each cardiometabolic indicator, and if there was a nonlinear relationship, piecewise linear regression was used to explore the dose-response relationship. Log-likelihood ratio test were used to compare the differences between the piecewise and linear model. Data analysis and RCS plotting were performed using SPSS 24.0 software and R-based language 4.1.0 version of R-studio software and Empowerstats software. *p* < 0.05 is considered statistically significant.

## 3 Results

### 3.1 Basic characteristics about participants

No participants dropped out halfway through the study, and a total of 77 participants were included in the statistical analysis, including 43 males and 34 females, aged 13.0 ± 1.7 years old, and the basic information is shown in Table 1.

**TABLE 1:** Basic characteristics of Participants.

	Total (*n* = 77)
Gender (boys/girls)	43/34
Age (years)	13.04 ± 1.70
Height (cm)	164.65 ± 7.98
Weight (kg)	84.53 ± 15.21
BMI (kg/m^2^)	30.89 ± 4.07
MVPA (min/day)	91.94 ± 36.92

BMI, body mass index; MVPA, moderate to vigorous physical activity.

### 3.2 Comparison of changes in CMR indicators before and after the intervention

WC, SBP, DBP, TC, TG, HDL-C, LDL-C, FINS, and HOMA-IR after intervention were significantly lower than those before intervention (*p* < 0.05). There was a decreasing trend in FPG, however, without statistical significance (*p* > 0.05). As shown in Table 2, there was a significant difference in the improvement of LDL-C among Q1∼Q3 groups (*p* < 0.05), and *post hoc* analysis revealed that the degree of improvement in the Q3 group was significantly higher than that in the Q1 groups (*p* < 0. 05).

**TABLE 2:** Comparison of the improvement degree of cardiometabolic risk indicators in different dose groups.

Outcomes	Q1 (≤ 79.4 min/day)	Q2 (79.4–112.4 min/day)	Q3 (>112.4 min/day)	*p*-value	Partial *η* ^*2*^
ΔBMI (kg/m^2^)	−2.52 ± 0.76	−2.76 ± 0.69	−2.65 ± 0.64	0.705	0.010
ΔWC (cm)	−7.08 ± 4.36	−7.59 ± 3.25	−7.04 ± 1.86	0.821	0.005
ΔSBP (mmHg)	−6.77 ± 8.26	−6.54 ± 9.83	−5.56 ± 7.75	0.233	0.039
ΔDBP (mmHg)	−7.38 ± 6.71	−5.96 ± 9.03	−4.64 ± 7.41	0.356	0.028
ΔTC (mmol/L)	−1.16 ± 1.52	−0.99 ± 1.04	−1.14 ± 0.97	0.639	0.012
ΔTG (mmol/L)#	−0.23 (−0.65, −0.07)	−0.09 (−0.18,0.11)	−0.37 (−0.62, −0.16)	0.060	−
ΔHDL-C (mmol/L)	−0.62 ± 0.21	−0.10 ± 0.20	−0.03 ± 0.26	0.549	0.016
ΔLDL-C (mmol/L)	−0.46 ± 0.25	−0.50 ± 0.38	−0.73 ± 0.42^a^	0.023	0.098
ΔFPG (mmol/L)	0.15 ± 0.94	−0.23 ± 1.14	−0.29 ± 1.08	0.137	0.006
ΔFINS (μU/mL)	−5.34 ± 7.00	−4.57 ± 9.18	−4.47 ± 7.78	0.961	0.001
ΔHOMA-IR#	−0.92 (−1.44, −0.25)	−0.82 (−1.30, −0.82)	−0.84 (−1.15, −0.02)	0.786	−

BMI, body mass index; DBP, diastolic blood pressure; FPG, fasting plasma glucose; FINS, fasting insulin; HOMA-IR, homeostasis model assessment index of insulin resistance; HDL-C, high-density lipoprotein cholesterol; LDL-C, low-density lipoprotein cholesterol; SBP, systolic blood pressure; TC, total cholesterol; TG, Triglycerides.

^a^ Indicates *p* < 0.05 compared with Q1 groups; #, indicates that the data is non-normally distributed, differences were compared using Kruskal-Wallis test. One-way analysis of covariance analysis corrected the baseline values of the indicators.

### 3.3 Dose-response relationship analysis between MVPA and improvement in CMR indicators

Linear regression was used to analyze the dose-response relationship between daily MVPA and improvement in CMR indicators, finding that for each 1 min/day increase in MVPA duration in the unadjusted model, ΔLDL-C decreased by 0.003 mmol/L [95% confidence interval (*CI*): −0.005, −0.001]. After adjusting for pre-test value, age, gender, and baseline BMI, ΔLDL decreased by 0.002 mmol/L (95% *CI*: −0.004, 0.001) for each 1 min/day increase in MVPA duration. In addition, no linear dose-response relationship was found between MVPA duration and other CMR indicators (*p* > 0.05, Table 3).

**TABLE 3:** Linear dose-response relationship analysis of MVPA and improvement in cardiometabolic risk indicators.

Outcomes	β(95%CI)	
	Model 1	Model 2
ΔWC (cm)	0.003 (−0.018, 0.024)	0.009 (−0.009, 0.028)
ΔSBP (mmHg)	0.028 (−0.060, 0.116)	0.036 (−0.013, 0.085)
ΔDBP (mmHg)	0.017 (−0.031, 0.065)	0.018 (−0.025, 0.061)
ΔTC (mmol/L)	−0.001 (−0.009, 0.006)	0.001 (−0.004, 0.004)
ΔTG (mmol/L)	−0.001 (−0.004, 0.002)	0.001 (−0.002, 0.002)
ΔHDL-C (mmol/L)	0.001 (−0.001, 0.002)	−0.001 (−0.001, 0.001)
ΔLDL-C (mmol/L)	−0.003 (−0.005, −0.001)*	−0.002 (−0.004, 0.001)
ΔFPG (mmol/L)	−0.003 (−0.010, 0.004)	−0.004 (−0.010, 0.001)
ΔFINS (μU/mL)	0.006 (−0.043, 0.055)	−0.013 (−0.043, 0.017)
ΔHOMA-IR	0.001 (−0.008, 0.008)	−0.004 (−0.012, 0.004)

BMI, body mass index; DBP, diastolic blood pressure; FPG, fasting plasma glucose; FINS, fasting insulin; HOMA-IR, homeostasis model assessment index of insulin resistance; HDL-C, high-density lipoprotein cholesterol; LDL-C, low-density lipoprotein cholesterol; SBP, systolic blood pressure; TC, total cholesterol; TG, Triglycerides. Model 1 = no adjustment; Model 2: adjusted for age, gender, baseline BMI, baseline value; *, *p* < 0.05 for model.

**FIGURE 1:**
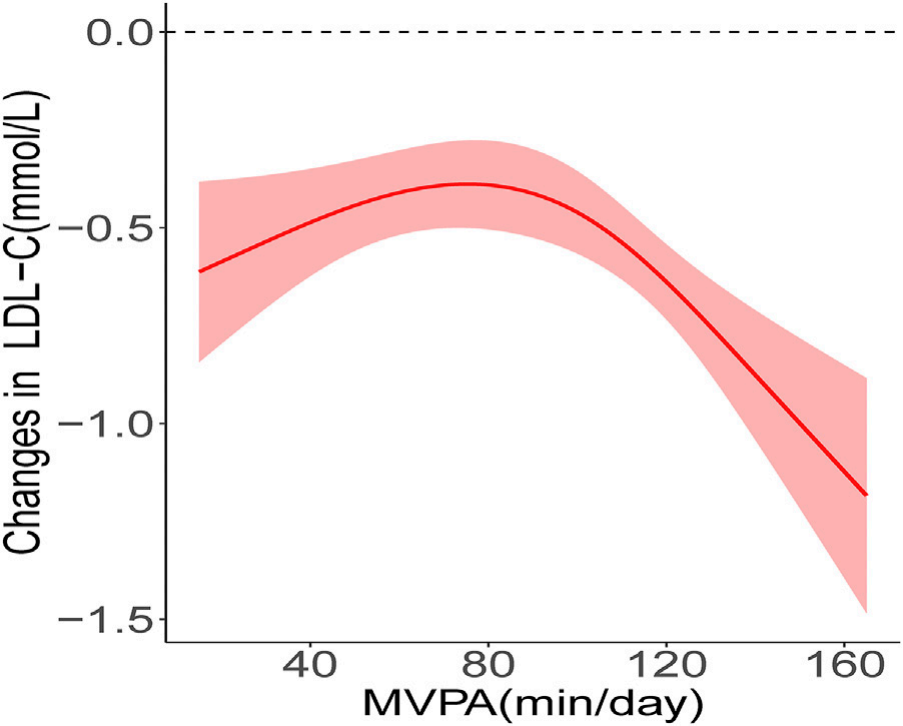
Nonlinear relationship between MVPA and LDL-C improvement. The red solid line shows the fitted curves, the red shaded areas show the 95% confidence intervals.

The results of the RCS analysis showed that there was a nonlinear relationship between MVPA and LDL-C improvement (*P*-Nonlinear < 0.05) and threshold effect (Figure 1), while the nonlinear dose-response relationship between MVPA and other indicators was not found (*P*-Nonlinear > 0.05) (Supplementary Figures S1–S9). The results of piecewise regression showed (Table 4) that when MVPA>77.1 min/day, increasing the daily MVPA duration resulted in higher improvement, and each 1 min/day increase in MVPA duration in the unadjusted model resulted in a 0.009 mmol/L (95% *CI*: −0.013, −0.005) decrease in ΔLDL-C. After adjusting for pre-test value, age, gender, and baseline BMI, ΔLDL-C decreased by 0.008 mmol/L (95% *CI*: −0.012, −0.004) for each 1 min/day increase in MVPA duration. When MVPA was ≤77.1min/day, increasing the daily MVPA duration did not increase the improvement of LDL-C.

**TABLE 4:** Nonlinear dose-response and threshold-effect relationship analysis of MVPA and LDL-C improvement.

	β(95%CI)	
	Model 1	Model 2
MVPA≤77.1 min/day	0.006 (0.001, 0.011)	0.006 (0.001, 0.011)
MVPA >77.1 min/day	−0.009 (−0.013, −0.005)	−0.008 (−0.012, −0.004)
*P* for model	<0.001	<0.001
*P* for likelihood ratio tests	<0.001	<0.001

BMI, body mass index; CI, confidence interval; MVPA, moderate to vigorous physical activity; Model 1 = no adjustment; Model 2: adjusted for age, gender, baseline BMI, baseline LDL-C.

## 4 Discussion

This study aimed to explore the dose-response relationship between daily MVPA duration and the improvement of CMR indicators in obese children and adolescents.

The results showed a non-linear dose-response relationship between daily MVPA duration and LDL-C improvement in obese children and adolescents. Only when MVPA is higher than 77.1 min/day, increasing the MVPA duration can obtain a higher LDL-C improvement effect. In clinical practice, WC is the evaluation indicator of abdominal obesity, SBP and DBP are the evaluation indicators of hypertension, and blood lipid level is used to judge whether dyslipidemia occurs, in addition, FPG, FINS and HOMA-IR are indicators of glucose metabolism (Li et al., 2022). All of these cardiometabolic risk indicators were associated with an increased risk of cardiovascular disease (CVD) (Park and Choi, 2022). Excessive accumulation of fat associated with obesity is an important cause of increased CMR (Yan et al., 2019). Previous studies have found that exercise intervention can effectively reduce the levels of weight, BMI, body fat percentage, TG, TC, LDL-C, FINS, SBP, DBP, and other CMR indicators in obese children and adolescents (Guo et al., 2011; Sigal et al., 2014; Liu et al., 2021). Similar to previous studies, the present study found significant improvements in BMI, WC, SBP, DBP, FINS, and lipids in obese children and adolescents after a 4-week exercise intervention. Notably, unlike previous studies (Wong et al., 2008; González-Ruíz et al., 2022; Meng et al., 2022) with longer intervention periods (12 weeks or longer), the obese children and adolescents in this study achieved significant health improvement after 4 weeks of exercise, suggesting that even short-period exercise interventions can achieve improved cardiometabolic health in obese children and adolescents. Elevated LDL-C is a necessary condition for atherogenesis induction, and no evidence of any clinically significant harm no matter how low the LDL-C level (Makover et al., 2022). Intervention such as exercise to reduce LDL-C levels in obese children and adolescents is crucial for the prevention of CVD. HDL-C is considered to be a cardiovascular risk protective factor, and higher HDL-C levels are associated with a lower risk of cardiovascular disease. The present study found a significant decrease in HDL-C levels after a 4-week exercise intervention, and similar results were observed in previous studies as well (Wang et al., 2011; Santos and Lavie, 2021). The reason for the HDL-C decrease may be related to the decrease in fat intake associated with dietary control during the intervention, as fatty acids are substrates of HDL-C (Santos and Lavie, 2021). Furthermore, Aicher et al. (Aicher et al., 2012) showed that although HDL-C levels decreased significantly after exercise intervention, cholesterol efflux capacity (CEC), an indicator reflecting the HDL-C’s ability of reverse cholesterol transport (RCT), did not change significantly, whereas CEC is considered to be an important indicator of HDL-C function (Rosenson, 2016) and its predictive value for the risk of cardiovascular disease is higher than that of low HDL-C (Khera et al., 2011). Therefore, this decrease in HDL-C due to the combination of exercise and dietary control is not associated with increased cardiovascular risk.

Since the 1990s, scholars have been studying the dose-response relationship between physical activity and health promotion to clarify the health effects of exercise doses produced by different combinations of exercise intensity, duration, and frequency (Haapanen et al., 1996; Kraus et al., 2001; Thompson et al., 2010). In the randomized controlled trial conducted by Davis et al. (Davis et al., 2012) on overweight/obese adolescents, the improvement of insulin sensitivity was more significant in participants of the high exercise dose group than in those of the low exercise dose group. Wu et al. (2021) found that increased levels of physical activity were associated with improved cardiometabolic risk, with a 0.359 mmol/L decrease in triglyceride and a 0.290 mmol/L increase in HDL-C for every 1METs increase in physical activity. Strizich et al. (Strizich et al., 2018) examined the relationship between objectively measured MVPA duration and cardiometabolic risk in 1,426 adolescents in a cross-sectional study and found that increased MVPA duration was associated with decreased triglycerides, and this relationship remained significant after adjustment for BMI and waist circumference. As with the previous studies mentioned above, the present study found the dose-response relationship between daily MVPA duration and improved CMR indicator in obese children and adolescents. In contrast to these previous studies, this paper found a significant nonlinearity in the dose-response relationship between MVPA and CMR indicator improvement, i.e., the improvement did not increase consistently with increasing MVPA duration. Several recent studies have also shown that the dose-response relationship between physical activity and health effects is not always linear. Sriram et al. (Sriram et al., 2021) found in a cross-sectional study consisting of normal weight adolescents that higher MVPA duration was not associated with lower SBP when the weekly MVPA duration exceeded 150 min and 90 min for boys and girls, respectively. Dipietro et al. (Dipietro et al., 2020) analyzed the relationship between different MVPA duration and the incidence of CMR aggregation in obese young adults and found that compared to participants with MVPA <150 min/week, those with MVPA between 150 and 300 min/week had a 66% lower incidence of CMR aggregation, whereas the incidence of CMR aggregation was only 61% lower in participants with MVPA >300 min/week, and the incidence of CMR aggregation did not decrease further with increasing MVPA time. In this study, we used RCS to analyze the dose-response relationship between the improvement of various CMR indicators (such as WC, SBP, DBP, FPG, TC, TG, etc.) and MVPA, but only the dose-response relationship between LDL-C and MVPA was found. As shown in Figure 1, the dose-response curve remained nearly stable at first, and then decreased rapidly after exceeding the threshold, reflecting two different trends of LDL-C improvement before and after the threshold point. The present study showed that when MVPA ≤77.1 min/day, increasing MVPA did not result in higher LDL-C improvement, while when MVPA >77.1min/day, a higher amount of MVPA resulted in more substantial improvement. Of course, not all previous studies are similar to the results of our study. Jenkins et al. (Jenkins et al., 2017) found that there was no dose-response relationship between the proportion of MVPA in the total time of physical activity and LDL-C level. The differences between the studies could be due to differences in indicators of physical activity, the participants’ weight status and ethnicity. In addition, the pre-post design used in our study also contributed to the difference between the results of previous cross-sectional studies.

Unlike previous studies, we illustrated the dose-response relationship between MVPA and CMR indicators in obese children and adolescents by pre-post quasi-experimental study design, and our findings might provide a certain reference for the development of precise exercise intervention strategies to improve cardiometabolic health in obese children and adolescents. This study has the following advantages: 1) it adopts the pre-post design with its control instead of a cross-sectional design, which avoids the limitation of cross-sectional design that cannot explore the causal relationship; and 2) it uses RCS to analyze whether there is a nonlinear relationship in the dose-response relationship, which avoids the limitation of simple linear regression that cannot explore the nonlinear relationship. Of course, this study has some limitations: 1) we only found a non-linear dose-response relationship between MVPA and LDL-C, but no dose-response relationship between other CMR indicators and MVPA was observed, which may be limited by the relatively short 4-week intervention period, and the intervention period should be extended for further study in the future; and 2) the participants were obese children and adolescents, and the closed centralized intervention led to difficulty in recruiting participants, thus limiting the further expansion of the sample size, which should be further expanded in the future for more far-reaching studies. 3) The absence of a control group may have affected the interpretation of the results to some extent.

## 5 Conclusion

There is a nonlinear dose-response relationship between MVPA and LDL-C improvement in obese children and adolescents. To achieve higher LDL-C improvement efficiency by increasing MVPA duration, daily MVPA duration should be higher than 77.1 min.

## Data Availability

The raw data supporting the conclusion of this article will be made available by the authors, without undue reservation.
