# Supplementary material for: Dose–response relationship between physical activity and cardiometabolic risk in obese children and adolescents: A pre-post quasi-experimental study
Source: Front Physiol. 2023 Jan 19;14:1070653. doi: 10.3389/fphys.2023.1070653 (PMC9892714; doi:10.3389/fphys.2023.1070653)
Supplement: Supplementary file 1 [file Table1.pdf]

Table S1 Sex-specific BMI cut-offs for screening overweight and obesity among school-aged children and adolescents aged 6 to 18 years

The unit of measurement is Kg/m^2

Age	Boy		Girl	
(year)	Overweight	Obesity	Overweight	Obesity
6.0 ~	16.4	17.7	16.2	17.5
6.5 ~	16.7	18.1	16.5	18.0
7.0 ~	17.0	18.7	16.8	18.5
7.5 ~	17.4	19.2	17.2	19.0
8.0 ~	17.8	19.7	17.6	19.4
8.5 ~	18.1	20.3	18.1	19.9
9.0 ~	18.5	20.8	18.5	20.4
9.5 ~	18.9	21.4	19.0	21.0
10.0 ~	19.2	21.9	19.5	21.5
10.5 ~	19.6	22.5	20.0	22.1
11.0 ~	19.9	23.0	20.5	22.7
11.5 ~	20.3	23.6	21.1	23.3
12.0 ~	20.7	24.1	21.5	23.9
12.5 ~	21.0	24.7	21.9	24.5
13.0 ~	21.4	25.2	22.2	25.0
13.5 ~	21.9	25.7	22.6	25.6
14.0 ~	22.3	26.1	22.8	25.9
14.5 ~	22.6	26.4	23.0	26.3
15.0 ~	22.9	26.6	23.2	26.6
15.5 ~	23.1	26.9	23.4	26.9
16.0 ~	23.3	27.1	23.6	27.1
16.5 ~	23.5	27.4	23.7	27.4
17.0 ~	23.7	27.6	23.8	27.6
17.5 ~	23.8	27.8	23.9	27.8
18.0 ~	24.0	28.0	24.0	28.0
