# Supplementary material for: Dose–response relationship between physical activity and cardiometabolic risk in obese children and adolescents: A pre-post quasi-experimental study
Source: Front Physiol. 2023 Jan 19;14:1070653. doi: 10.3389/fphys.2023.1070653 (PMC9892714; doi:10.3389/fphys.2023.1070653)
Supplement: Supplementary file 2 [file Image1.pdf]

Restricted cubic spline plot of MVPA and cardiometabolic risk indicators

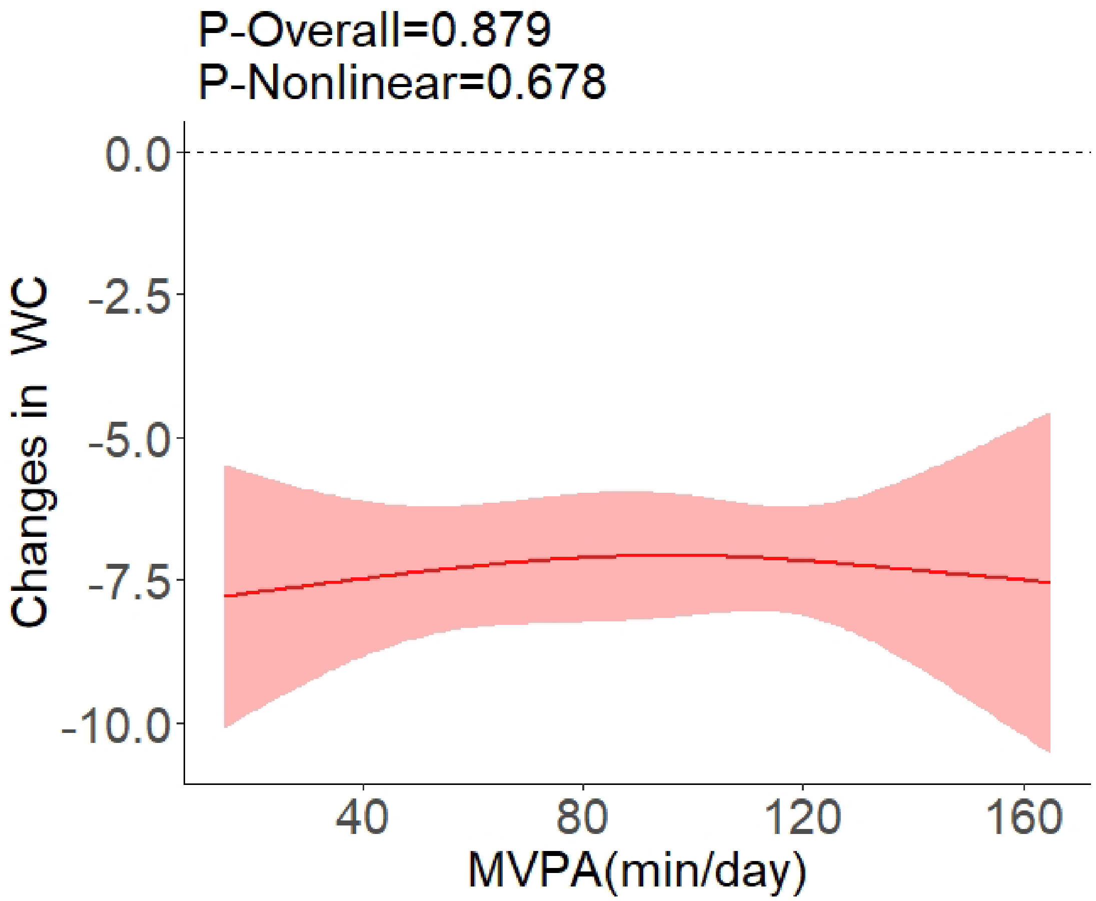

Figure S1 Restricted cubic spline plot of MVPA with changes in WC

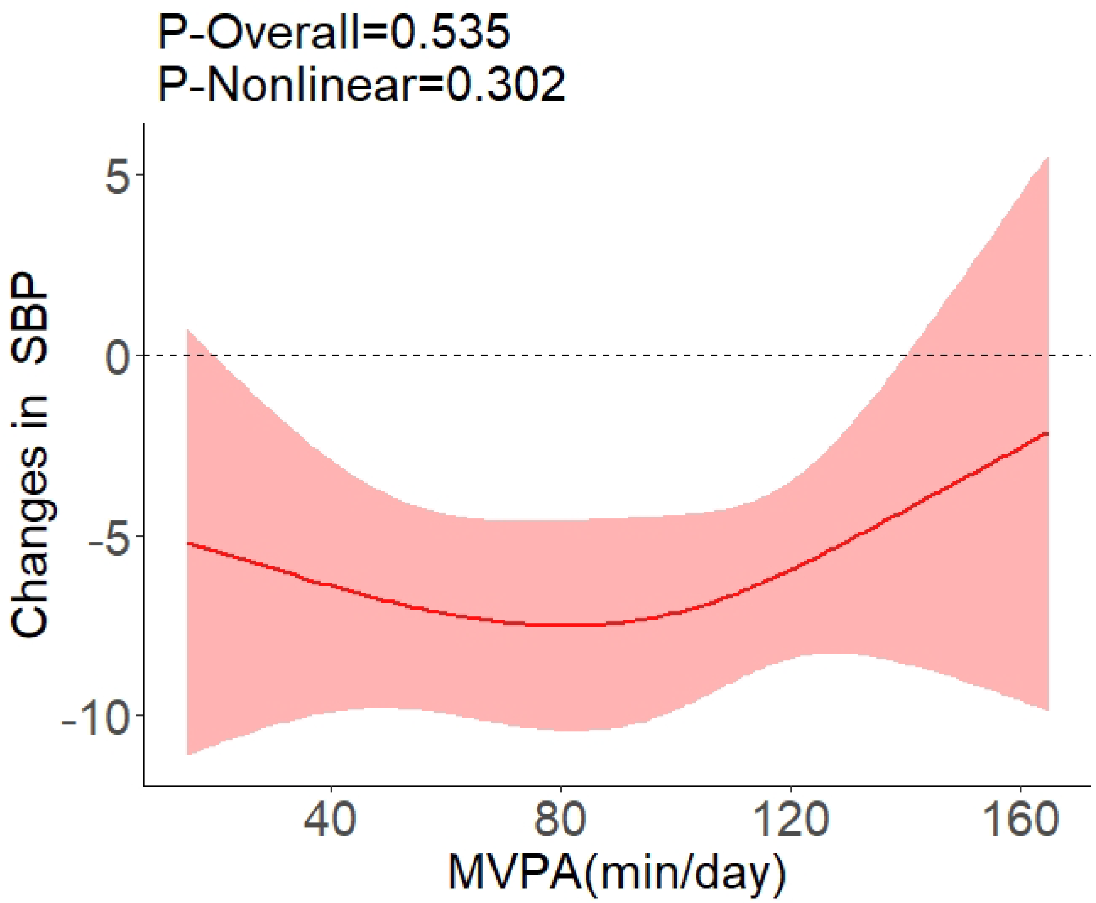

Figure S2 Restricted cubic spline plot of MVPA with changes in SBP

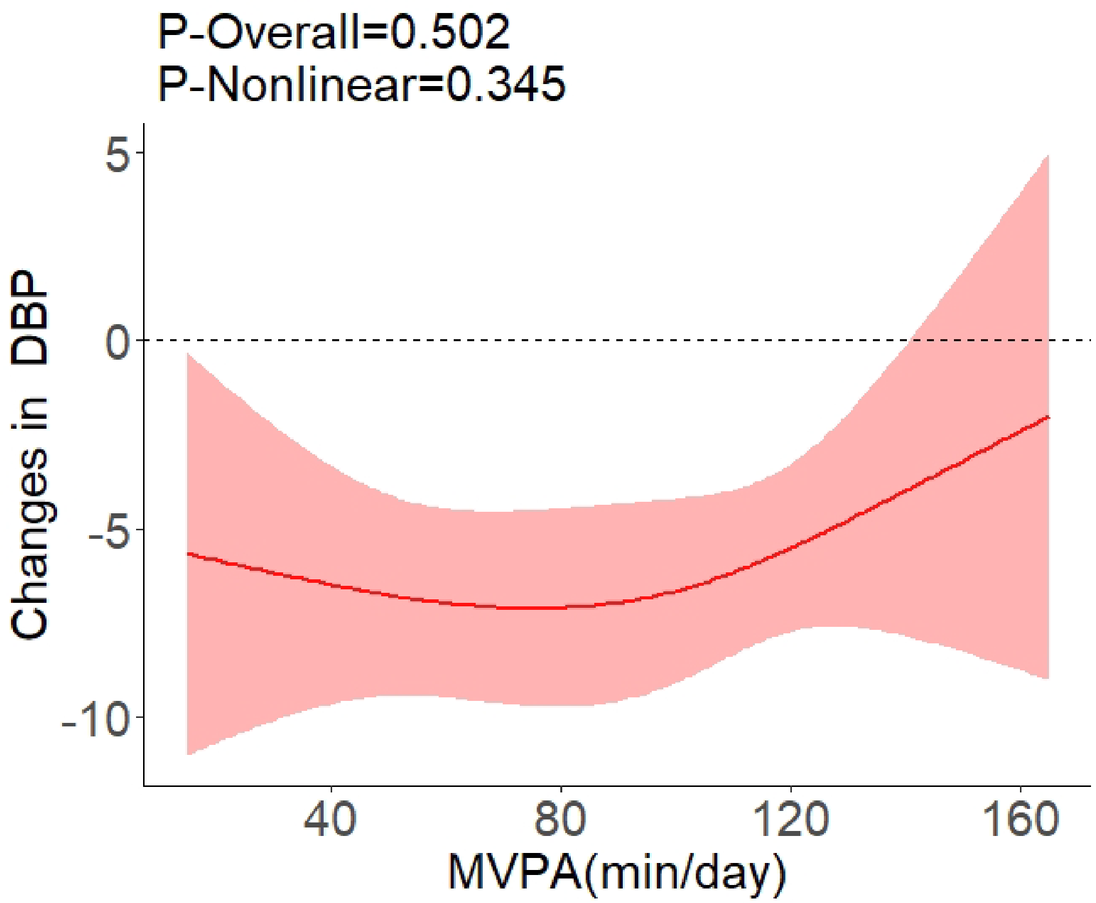

Figure S3 Restricted cubic spline plot of MVPA with changes in DBP

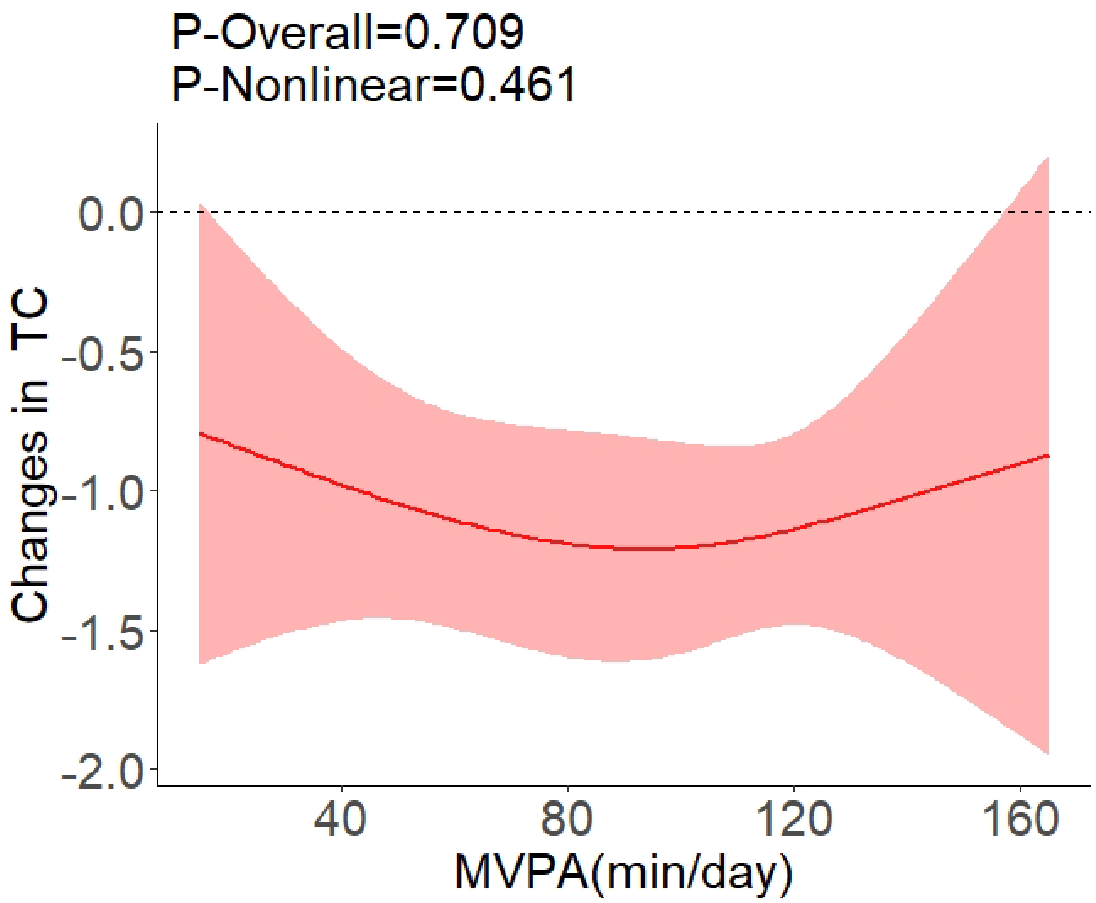

Figure S4 Restricted cubic spline plot of MVPA with changes in TC

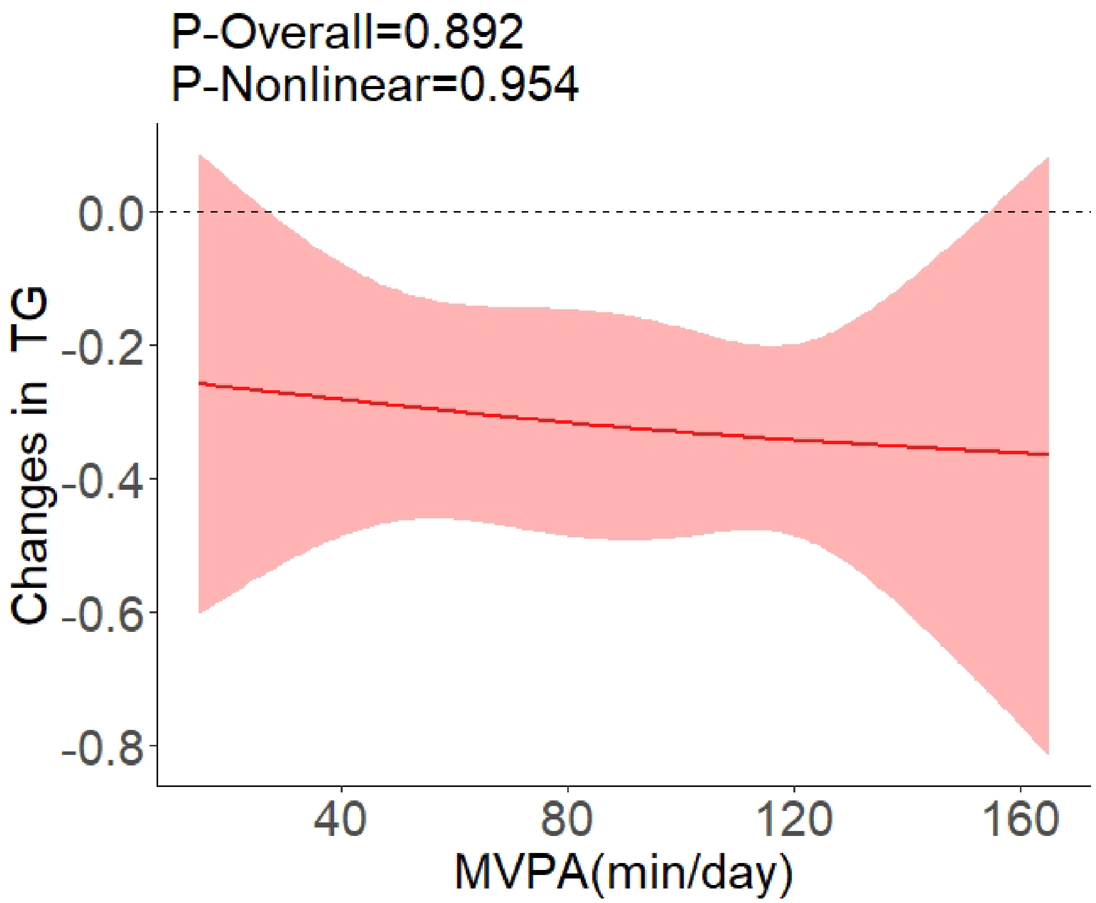

Figure S5 Restricted cubic spline plot of MVPA with changes in TG

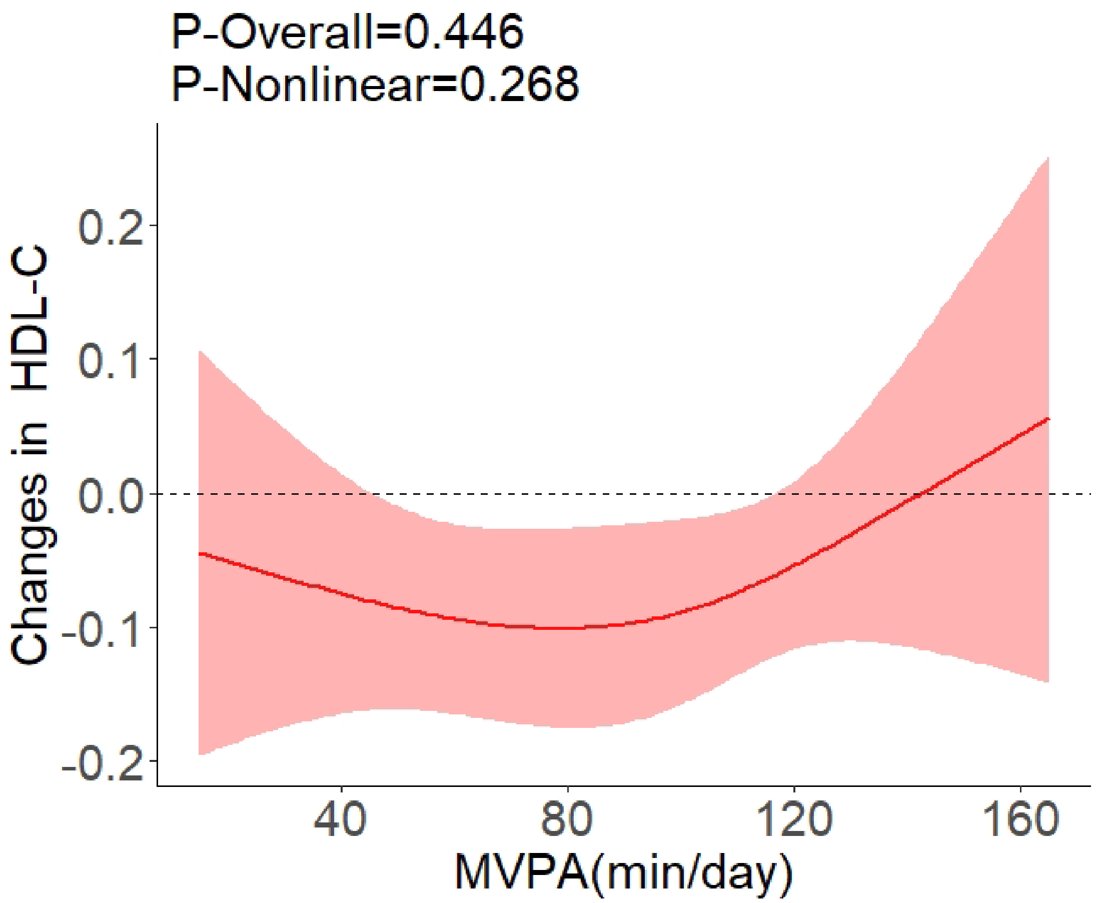

Figure S6 Restricted cubic spline plot of MVPA with changes in HDL-C

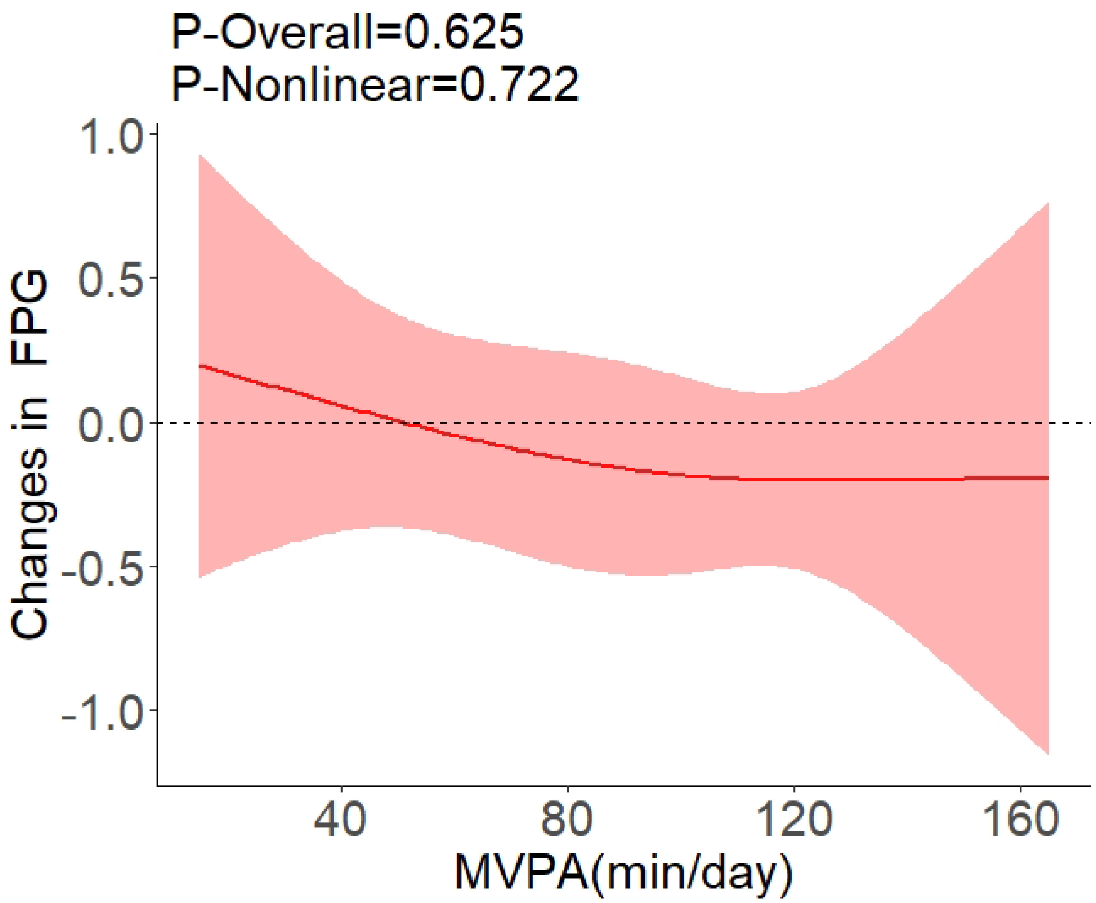

Figure S7 Restricted cubic spline plot of MVPA with changes in FPG

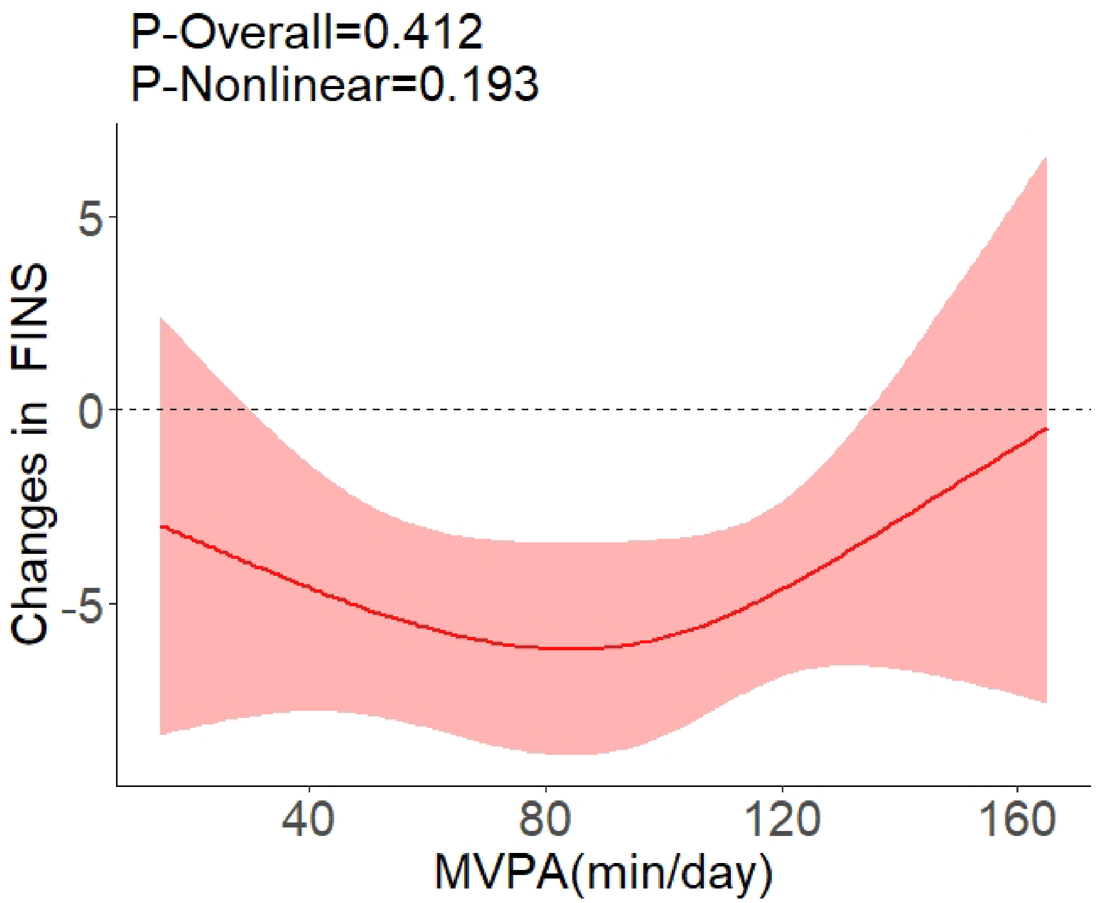

Figure S8 Restricted cubic spline plot of MVPA with changes in FINS

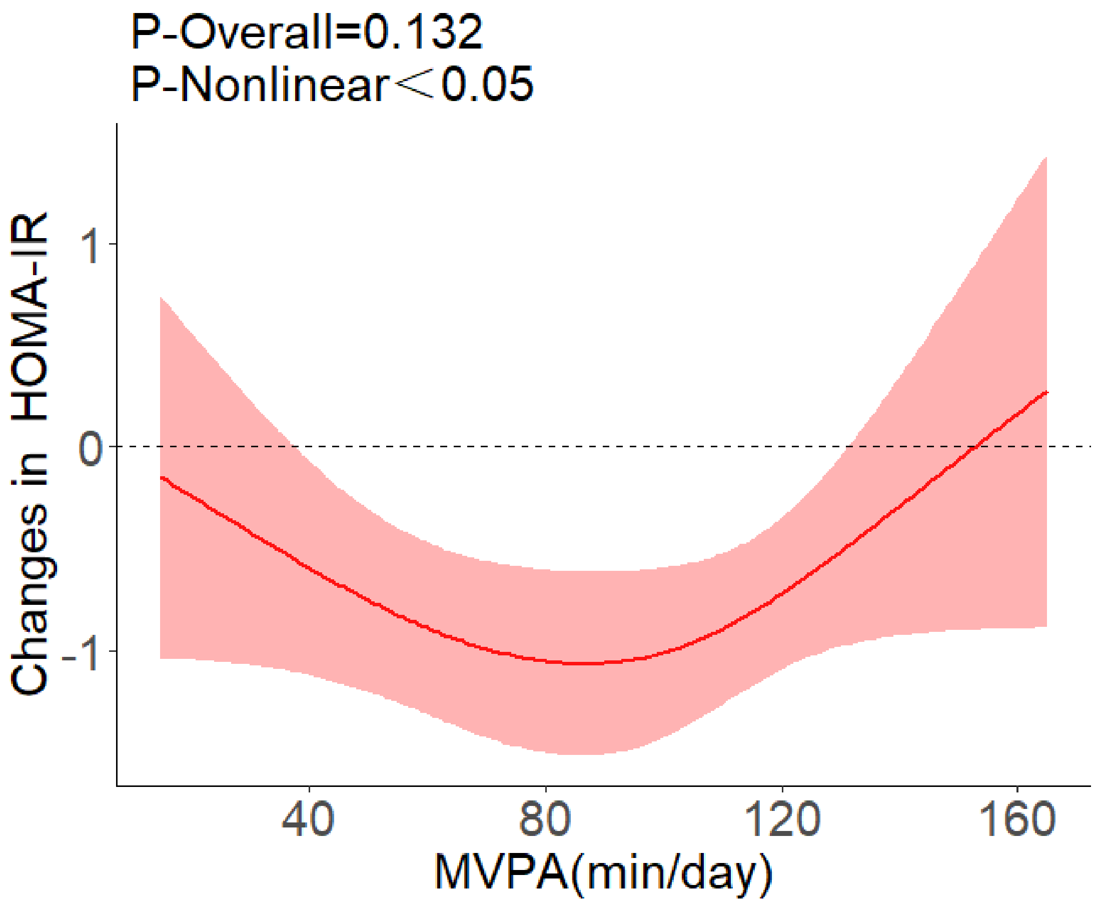

Figure S9 Restricted cubic spline plot of MVPA with changes in HOMA-IR
